# 

**Published:** 1882-10

**Authors:** 


### CONTENTS.

| | Page. | | Page. |
|---|---|---|---|
| Quenching Thirst | 465 | Poetry | 476 |
| How People Take Cold | 466 | The Dying | 477 |
| Digestion | 467 | Hygiene | 478 |
| Running Up Stairs | 468 | Health of Employments | 483 |
| Sugar and Teeth | 469 | Sudden Death | 487 |
| Tobacco | 469 | Longevity | 489 |
| Perils of Flattery | 472 | Checking Perspiration | 490 |

